# Supplementary material for: Adoption and implementation of teleaudiology as a telehealth model in Jordan and Arab countries: A cross-sectional survey
Source: PLoS One. 2026 Mar 3;21(3):e0343923. doi: 10.1371/journal.pone.0343923 (PMC12956131; doi:10.1371/journal.pone.0343923)
Supplement: Attachment — Submitted filename: Reviewers comment.docx [file pone.0343923.s001.docx]

Reviewers comment:

**Abstract:**

- The abstract is structured and clearly reports background, methods, key results, and conclusions, allowing readers to quickly understand the scope of the study.
- Temper the conclusion by explicitly stating that findings reflect perceptions rather than effectiveness or outcomes.

**Introduction**

- The introduction provides a comprehensive overview of teleaudiology, supported by relevant and up-to-date international literature.
- The introduction is overly long and reads more like a narrative review than a focused rationale for the present study.
- Repetition is evident when discussing COVID-19–related acceleration of telehealth adoption. Methodological limitations of previous studies are not sufficiently highlighted. Add critical comparisons between studies rather than descriptive summaries.
- The transition from global literature to the Arab-region context is gradual and lacks a strong problem statement. Add a concise paragraph clearly identifying the research gap and problem statement.
- Research questions or hypotheses are not explicitly articulated.

**Methods**

- Ethical approval, participant consent, and statistical procedures are clearly documented, enhancing transparency and ethical rigor.
- No validation process for the adapted questionnaire is described beyond internal consistency. Describe content or face validity procedures for the survey also the content validity Index (CVI) for the questionnaire used.
- The rationale for grouping providers vs. non-providers is not explained in the study.
- Country-wise sample imbalance (e.g., dominance of Jordan) is not addressed.
- Potential response bias due to online distribution of the questionnaire is not discussed.

**Results**

- Results are detailed, systematically presented, and supported by tables and figures that enhance clarity.
- The results section is excessively long and contains interpretative statements better suited for the discussion.
- Some statistical findings (e.g., extremely small odds ratios) are reported without sufficient explanation.
- Multiple analyses appear exploratory rather than hypothesis-driven.
- Redundancy exists between text and tables, leading to repetition.
- Clinical relevance of statistically significant findings is not clearly discussed.
- Phrase Non-providers in line 299 –change it to non-providers of tele audiology in practise

**Discussion**

- The discussion effectively integrates current findings with existing international and regional literature.
- Shift focus from restating results to interpreting their significance.

**Conclusion**

- The conclusion succinctly summarises the main findings and reinforces the relevance
- The conclusion introduces ideas (e.g., equity and sustainability) not fully developed earlier. Align conclusions more strictly with the study design and data. Provide 2–3 clearly prioritised, actionable recommendations based on the present study findings.

**General Comments:**

Remove figures that merely restate tabular data unless they add interpretive value.

**Figure 1:** The figure title is misleading; it implies comparison by experience, but the figure is referenced in the text in relation to timing of adoption.

**Figure 2:** Percentages can be clearly labelled on each bar. Consider grouping services into broader categories (e.g., diagnostic vs rehabilitative).

**Figure 3:** The comparative approach (most vs least effective) adds interpretive value beyond simple frequency counts. The figure does not clarify whether effectiveness is based on clinical outcomes, feasibility, or user satisfaction.
